# Metabolism of Soy Isoflavones by Intestinal Bacteria: Genome Analysis of an *Adlercreutzia equolifaciens* Strain That Does Not Produce Equol

**DOI:** 10.3390/biom10060950

**Published:** 2020-06-23

**Authors:** Lucía Vázquez, Ana Belén Flórez, Begoña Redruello, Baltasar Mayo

**Affiliations:** 1 Departamento de Microbiología y Bioquímica, Instituto de Productos Lácteos de Asturias (IPLA), Consejo Superior de Investigaciones Científicas (CSIC), 33300 Villaviciosa, Spain; lucia.vazquez@ipla.csic.es (L.V.); abflorez@ipla.csic.es (A.B.F.); 2 Instituto de Investigación Sanitaria del Principado de Asturias (ISPA), 33011 Oviedo, Spain; 3 Servicios Científico-Técnicos, Instituto de Productos Lácteos de Asturias (IPLA), Consejo Superior de Investigaciones Científicas (CSIC), 33300 Villaviciosa, Spain; bredruel@ipla.csic.es

**Keywords:** soy isoflavones, daidzein, genistein, equol, *Adlercreutzia equolifaciens*, intestinal microorganisms, fecal microbiota

## Abstract

Isoflavones are transformed in the gut into more estrogen-like compounds or into inactive molecules. However, neither the intestinal microbes nor the pathways leading to the synthesis of isoflavone-derived metabolites are fully known. In the present work, 73 fecal isolates from three women with an equol-producing phenotype were considered to harbor equol-related genes by qPCR. After typing, 57 different strains of different taxa were tested for their ability to act on the isoflavones daidzein and genistein. Strains producing small to moderate amounts of dihydrodaidzein and/or *O*-desmethylangolensin (*O*-DMA) from daidzein and dihydrogenistein from genistein were recorded. However, either alone or in several strain combinations, equol producers were not found, even though one of the strains, W18.34a (also known as IPLA37004), was identified as *Adlercreutzia equolifaciens*, a well-described equol-producing species. Analysis and comparison of *A. equolifaciens* W18.34a and *A. equolifaciens* DSM19450^T^ (an equol producer bacterium) genome sequences suggested a deletion in the former involving a large part of the equol operon. Furthermore, genome comparison of *A. equolifaciens* and *Asaccharobacter celatus* (other equol-producing species) strains from databases indicated many of these also showed deletions within the equol operon. The present results contribute to our knowledge to the activity of gut bacteria on soy isoflavones.

## 1. Introduction

The consumption of soy and soy-derived products correlates with better intestinal health, reduced menopause symptoms, and a smaller prevalence of hormone-mediated syndromes, cardiovascular disease, and cancer (for a recent review, see Zaheer et al. [1]). Soy has many biologically active compounds [2], but its beneficial health effects have been repeatedly attributed to its isoflavone content [3]. Isoflavones are polyphenols, the chemical structure of which resembles that of 17-β-oestradiol [4]; this invests these molecules with hormonal-like activity [5]. As recorded for other polyphenols, isoflavones also have antioxidant [6] and enzyme-inhibitory [7] properties. All of these features may contribute to their supposed health benefits.

Dietary isoflavones are sequentially transformed into their active metabolites by cellular enzymes and enzymes from the gut microbiota [3]. Cellular and bacterial glycosyl hydrolases release isoflavone–aglycones from the isoflavone–glycosides present in plants [8]. Aglycones are more bioavailable and active than their glycoside counterparts [1,8], and can be further processed, undergoing dihydroxylation, reduction, breakage of the pyrone ring, or demethylation, etc., to produce compounds that either possess greater estrogenic activity (such as equol) or that are inactive (such as *O*-desmethylangolensin; *O*-DMA) [9]. However, while 80–90% of people can produce *O*-DMA, only 25–50% of humans produce equol [10,11,12]. Both *O*-DMA and equol are produced from the isoflavone daidzein via the exclusive action of intestinal bacteria [9].

Our knowledge of the species and pathways involved in the synthesis of the above and other isoflavone-derived metabolites remains scarce [5,13]. Improving is important since it may well be that only those persons able to produce certain metabolites such as equol may benefit fully from the consumption of soy or isoflavones [14,15]. In recent decades, several equol-producing strains of bacteria have been identified and characterized [16]. Most of them are anaerobic species belonging to the phylum *Actinobacteria*, class *Coriobacteriia*, family *Eggerthellaceae* [17]. Certainly, strains of *Adlercreutzia equolifaciens* [18], *Asaccharobacter celatus* [19], *Enterorhabdus mucosicola* [20], *Slackia isoflavoniconvertens* [21], *Slackia equolifaciens* [22], and other related bacteria have been reported to be equol producers. Equol biosynthesis in these strains takes place through dihydrodaidzein and tetrahydrodaidzein intermediates via a process involving three reductases [23,24,25,26]. Some equol-producing bacteria have also been shown to intervene in the conversion of genistein to 5′-hydroxy-equol [27], a compound with a chemical structure similar to equol that might have comparable properties. However, it remains unknown whether other intestinal microbes are involved in isoflavone metabolism and equol formation [9], and this prevents us from developing strategies for modulating the endogenous production of isoflavone-bioactive metabolites via, for example, the use of prebiotics, probiotics, or other dietary supplements [28,29]. Furthermore, since bacterial equol and 5′-hydroxy-equol producers are fastidious and extremely oxygen-susceptible, well-characterized strains of more manageable species might be better suited to the large-scale biotechnological production of these active derivatives.

The aim of the present work was to contribute to the knowledge of isoflavone metabolism by intestinal bacteria. To this end, isolated colonies from fecal samples belonging to three women that produced equol were subjected to real-time quantitative PCR (qPCR) analysis, targeting the gene encoding the tetrahydrodaidzein reductase (*tdr*) required for equol biosynthesis. Presumptive positive isolates were then identified and typed by molecular methods, and the activity of different strains on daidzein and genistein was tested after culturing in the presence of these isoflavones. Finally, isoflavones and isoflavone-derived metabolites in the cultures were identified and quantified by ultra-high-performance liquid chromatography (UHPLC). In addition, a single recovered isolate of the species *A. equolifaciens* that did not produce equol from daidzein was subjected to genome sequencing and analysis, and its genome was then compared to those of closely related species available in databases.

## 2. Materials and Methods

### 2.1. Bacteria and Culture Conditions

This study was approved by the Ethical Research Committee of Asturias Principality, Spain, with reference number 84/14. Intestinal bacteria were isolated from dilutions of fresh stool samples donated by three women with an equol-producing status (W3, W8, and W18) identified in a previous work [30]. Before sampling, volunteers signed a written informed consent. Variable amounts of dihydrogenistein, dihydrodaidzein, and equol from genistein and daidzein, respectively, were measured in fecal cultures inoculated with the same fecal samples utilized in this work as a source of microorganisms [30]. Dilutions of the samples were plated on Gifu Anaerobic Medium (GAM; Nissui, Tokyo, Japan) agar supplemented with 0.5% arginine (Merck, Darmstad, Germany), thus producing GAM-Arg plates, and incubated at 37 °C for up to five days under anoxic conditions (10% H_2_, 10% CO_2_, 80% N_2_) in a Mac500 work-station (Don Whitley, Bingley, UK). Though non-selective, GAM-Arg is suitable for growing equol producer strains of several species [30]. Colonies were collected daily and purified twice on the same plates. They were then inoculated into liquid GAM-Arg and stored at −80 °C with 15% glycerol (Merck). *Adlercreutzia equolifaciens* DSM 19450^T^, *Asaccharobacter celatus* DSM 18785^T^, *Enterorhabdus mucosicola* DSM 19490^T^*, Slackia equolifaciens* DSM 24851^T^, and *Slackia isoflavoniconvertens* DSM 22006^T^ were obtained from the German Collection of Microorganisms and Cell Cultures (DSMZ, Braunschweig, Germany), cultured under the above conditions, and used as equol-producing controls.

### 2.2. Quantitative Real-Time PCR (qPCR)

Stored isolates were recovered on GAM-Arg agar plates and cell-free extracts from single colonies, obtained as described by Ruiz-Barba et al. [31] with minor modifications, and used in qPCR amplifications. Briefly, colonies were suspended in 100 μL of molecular-biology-grade water (Sigma-Aldrich, St. Louis, CA, USA), and subjected to heat treatment at 98 °C for 30 min. An equal volume of chloroform/isoamyl alcohol (24:1) (Sigma-Aldrich) was added and the cell suspensions vortexed for 5 s and then centrifuged at 16,000× *g* for 5 min. The upper aqueous phase was used as a source of DNA in the qPCR assays, all performed in a 7500 Fast Real-Time PCR System running proprietary software v.2.0.4 (Applied Biosystems, Foster City, CA, USA). The qPCR was accomplished by using a primer pair targeting the *tdr* gene, which encodes a tetrahydrodaidzein reductase involved in equol production [30]. Briefly, reactions were performed in a final volume of 20 μL containing 10 μL of a 2xSYBR Green PCR Master Mix with ROX as a passive reference (Applied Biosystems), 900 nM of each primer, and 5 μL of cell-free extract. The standard amplification protocol consisted of an initial cycle at 95 °C for 10 min, followed by 40 cycles at 95 °C for 15 s, and 1 min at 60 °C. After amplification, the melting curves were analyzed and compared to those obtained with total DNA purified from the fecal samples of the women and that of the equol-producing bacterial controls.

### 2.3. Identification of Bacteria

Isolates with a presumptive positive qPCR result were identified after isolation of their total DNA using the GenElute Bacterial Genomic DNA Kit (Sigma-Aldrich). To this end, the 16S rRNA gene was amplified using the universal oligonucleotide primers 27F (5′-AGAGTTTGATCCTGGCTCAG-3′) and 1492R (5′-GGTTACCTTGTTACGACTT-3′). The PCR conditions were as follows: one cycle at 95 °C for 5 min, 35 cycles at 94 °C for 30 s, 55 °C for 45 s, and 72 °C for 2 min, and a final extension cycle at 72 °C for 10 min. PCR products were subjected to electrophoresis in 2% agarose gels, stained with ethidium bromide (0.5 µg/mL), and visualized under UV light using a G Box Chemi XRQ gel doc system (Syngene International, Bangalore, India). Amplicons were then purified using GenElute PCR Clean-Up columns (Sigma-Aldrich) and sequenced at a sequencing service (Macrogen, Madrid, Spain). Sequences were then compared to those in the GenBank database using the BLAST+ software 2.10.0 version [32], and in the Ribosomal Database Project database Release 11 using the Classifier tool [33].

### 2.4. Typing of Isolates

Isolates were genotyped according to their combined RAPD- and rep-PCR fingerprinting profiles using primer M13 (5′-GAGGGTGGCGGTTCT-3′) as reported by Rossetti and Giraffa [34], primer BoxA2R (5′-ACGTGGTTTGAAGAGATTTTCBG-3′) as reported by Koeuth et al. [35], and primer OPA18 (5′-AGGTGACCGT-3′) as reported by Mättö et al. [36]. PCR amplifications were independently performed in 25 μL volume reactions containing 12.5 μL MasterMix (Ampliqon), 5 μL of primer (10 μM), 3 μL of purified DNA, and molecular-grade water. The DNA amplification conditions were as follows: one cycle of 95 °C for 7min, 40 cycles of denaturation at 90 °C for 30 s, primer annealing for 1 min at 42 °C for M13, 40 °C for BoxA2R, or 32 °C for OPA18, an extension at 72 °C for 4 min, and a final extension step at 72 °C for 10 min. Amplicons were electrophoresed and visualized as above. GeneTools software v.4.03 (SynGene, Cambridge, UK) was used to compare and cluster the profiles using the unweighted pair group with the arithmetic mean (UPGMA) method. The similarity of patterns was expressed via simple matching (SM) coefficients. The results of triplicate typing analyses were 94% reproducible; profiles with ≥94% similarity were thus considered to be the same strain.

### 2.5. Detection and Quantification of Isoflavones and Isoflavone Metabolites

Daidzein, genistein, and their derived metabolites dihydrodaidzein, dihydrogenistein, *O*-DMA, and equol were detected and quantified by UHPLC based on the method for isoflavone determination in urine samples reported by Redruello et al. [37]. Briefly, the control strains were independently cultured in GAM-Arg medium supplemented with 12.5–100 µM daidzein or genistein (LC Laboratories, Woburn, MA, USA). Furthermore, the selected strains were inoculated in pairs, triads, and tetrads and cultured with 100 µM of each isoflavone as above. After overnight incubation, cultures were centrifuged at 16,000× *g* for 2 min, and then filtered through a 0.2 µm polytetrafluoroethylene (PTFE) membrane (VWR, Radnor, PA, USA). The culture supernatants were used directly in UHPLC analyses. Quantification was performed against calibration curves for isoflavone and isoflavone-derived standards obtained from a commercial source (LC Laboratories). In this work, the limit of quantification (LoQ) for the different compounds analyzed were, in µM, 6.25 for daidzein, genistein, and dihydrogenistein, 5.62 for *O*-DMA, 3.13 for dihydrodaidzein, and 3.12 for equol.

### 2.6. Genome Analysis of Adlercreutzia equolifaciens W18.34a

DNA and deduced protein sequences from the genome of *A. equolifaciens* W18.34a (also known as IPLA 37004; [38]) were examined individually for homology against non-redundant DNA and protein databases using BLAST software (BLASTn and BLASTp, respectively) as above. To visualize the diversity and the evolutionary relationships between *Coriobacteriia* species, the genome sequences of type strains in GenBank were downloaded, aligned, and compared to that of W18.34a. A phylogenetic tree was created using the phylogenetic tree building service using PATRIC v.3.6.3 software [39] employing the “Codon Tree” workflow and the genome sequence of *Bifidobacteirum longum* subsp. *infantis* DSM 20,088 (GenBank NC_011593.1) as an outgroup. Briefly, alignments were performed against 100 shared protein sequences from the PATRIC global protein families (PGFams) using Muscle software [40]; nucleotide sequences were compared using the *codonalign* function in BioPython [41]. A concatenated alignment of all proteins and nucleotides was generated and visualized using Randomized Axelerated Maximum Likelihood (RAxML) [42] and FigTree software v. 1.4.3 (http://tree.bio.ed.ac.uk/software/figtree/), respectively. Complementarily, genome sequences of all strains in GenBank belonging to *A. equolifaciens* and to the closely related species *As. celatus* were aligned and compared to the W18.34a genome using Mauve software v. 2.4.0 [43] and Vector NTI (Thermo Fisher Scientific, Waltham, MA, USA) programs.

## 3. Results

More than 500 colonies from the dilutions of the fecal samples were screened by qPCR targeting the *tdr* gene (involved in the synthesis of equol). Analysis of the melting curves for 73 isolates suggested that these organisms might contain the target or a related gene. Despite this similarity in the melting curves, the Ct of the reactions was, in all cases, higher than 30, the limit of detection of the qPCR assay established in the previous work [30], suggesting this may represent a negative result. The molecular identification of the 73 isolates showed that they belonged to four distinct phyla, were grouped into 10 families, and belonged to 21 species-related taxa, of which the most abundant were *Eggerthella lenta* (19 isolates), *Escherichia coli* (17), *Collinsella* spp. (10), *Bifidobacterium* spp. (8), and *Anaerococcus* spp. (4) (Table 1). In addition, one of the isolates, W18.34a, was identified as *A. equolifaciens*, a well-known equol-producing species [18]. Under the experimental RAPD and rep-PCR typing conditions, 57 different strains were deemed detected among the 73 isolates (Figure S1).

To establish appropriate conditions for analyzing isoflavone metabolism, the control strains were incubated with varying concentrations of daidzein (12.5 to 100 µM) for 24 and 48 h (Table 2). Daidzein was rapidly used by all strains under most conditions; however, the synthesis of equol varied widely among the strains. At 24 h of incubation, S. *isoflavoniconvertens* DSM22006^T^ and *A. equolifaciens* DSM 19450^T^ transformed all daidzein into equol under all tested concentrations of daidzein, while *S. equolifaciens* DSM 24851^T^ completed the transformation only when a concentration of 100 µM was used. The production of equol from daidzein by *S. isoflavoniconvertens* DSM22006^T^ (Table 2 and Table 3), and occasionally by *A. equolifaciens* DSM 19450^T^, reflected values higher than expected for the amount of daidzein added (Table 2). Smaller amounts of equol were always obtained with *As. celatus* DSM 18785^T^ and *E. mucosicola* DSM 19490^T^. The concentration of equol was always higher at 24 than at 48 h, suggesting this compound is either further transformed or degraded by these strains under prolonged culturing. Based on these results, 100 µM isoflavone and 24 h incubation time were selected to test the fecal strains.

All 57 strains were assayed for isoflavone metabolism in GAM-Arg medium supplemented with either 100 µM of daidzein or genistein. Table 3 summarizes the results obtained. Between 40 and 100% of the isoflavones added to the medium were recovered from the cultures as (correspondingly) unaltered daidzein or genistein. Isoflavone derived metabolites were clearly detected in some cultures even though the values obtained were occasionally below the limit of quantification. In the cultures with daidzein, low values of dihydrodaidzein (~3 µM) and/or *O*-DMA (~10 µM) were quantified in supernatants from some isolates of different bacterial lineages. The latter compound was mostly produced by members of the class *Coriobacteriia*, which includes species belonging to the families *Coriobacteriaceae* and *Eggerthellaceae*. Whenever a chromatographic peak was detected at the elution position of equol, the concentration was always below the LoQ for this compound (3.12 µM). This prompted all the analyzed strains to be deemed equol non-producers. As the original fecal samples produced equol but none of our isolates did, strains of different species were combined (in groups of two up to four) to test whether equol production was the result of complementary activities found in different microbes. Under the same culture conditions, no equol was detected when strain mixtures were grown together. Low levels of dihydrogenistein (3–7 µM) were detected in the supernatants of isolates from species such as *Escherichia coli* (seven strains) and *E. lenta* (four strains). The highest dihydrogenistein concentrations were detected in the supernatant of two *Bifidobacterium adolescentis* strains (10–16 µM). After incubation, the *S. isoflavoniconvertens* DSM22006^T^ control strain converted about 20% of the genistein into dihydrogenistein.

**Figure 1:**
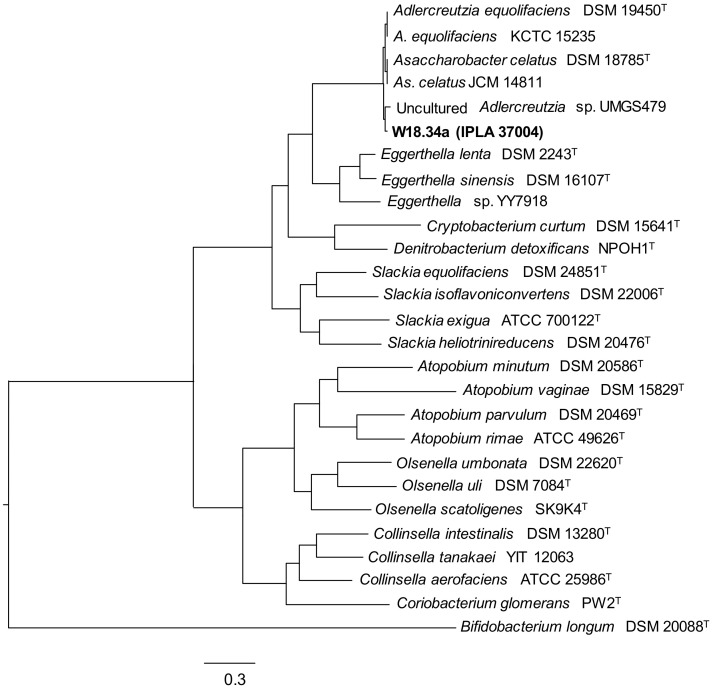
Phylogenetic tree obtained by concatenated alignment of all proteins and genes from the genome sequence of *Adlercreutzia equolifaciens* W18.34a and other *Coriboacteriia* species and strains. The genome sequence of *Bifidobacterium longum* DSM 20088^T^ was used as an outgroup.

**Figure 2:**
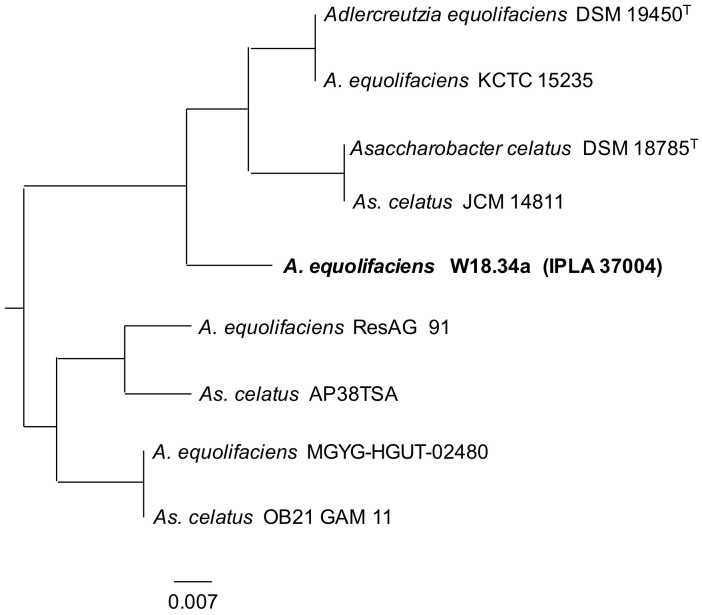
Phylogenetic relationships of *Asaccharobacter celatus* and *Adlercreutzia equolifaciens* strains (including W18.34a; IPLA 34007) obtained by comparison of their genome sequences.

Surprisingly, strain W18.34a, identified as belonging to *A. equolifaciens*, produced some *O*-DMA from daidzein (about 10%), but did not produce any equol. This prompted the sequencing of its genome, recorded as IPLA37004 in the GenBank database (Assembly entry GCA_009874275.1) [38]. Phylogenomic analysis based on concatenated single-copy core-genome proteins and genes assigned W18.34a to a branch with *A. equolifaciens* and *As. celatus* strains (Figure 1): strains of the biotypes reported to produce equol. It should be noted, however, that these two species were described at around the same time [18,19], suggesting, as recently proposed, that they might still represent the same taxon [17]. Phylogenomic analysis of all *A. equolifaciens* and *As. celatus* strains in the NCBI database [44] comparing concatenated genome sequences reinforced this possibility (Figure 2).

**Figure 3:**
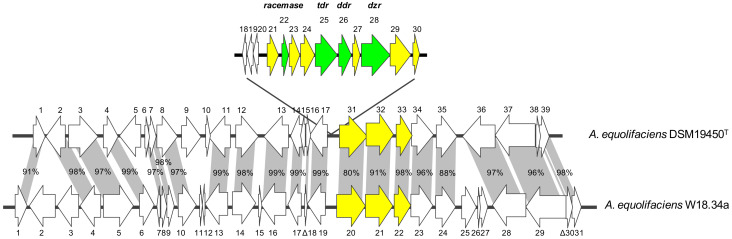
Diagram showing the genetic organization of upstream and downstream ORFs flanking the equol gene cluster in *A. equolifaciens* DSM19450^T^ and *A. equolifaciens* W18.34a, and the percentage of amino acid identity of the deduced proteins encoded by shared genes. In green, genes coding for the well-known proteins involved in equol biosynthesis racemase (*racemase*), daidzein reductase (*dzr*), dihydrogenistein reductase (*ddr*), and tetrahydrodaidzein reductase (*tdr*); in yellow, other genes within the equol biosynthesis operon.

**Figure 4:**
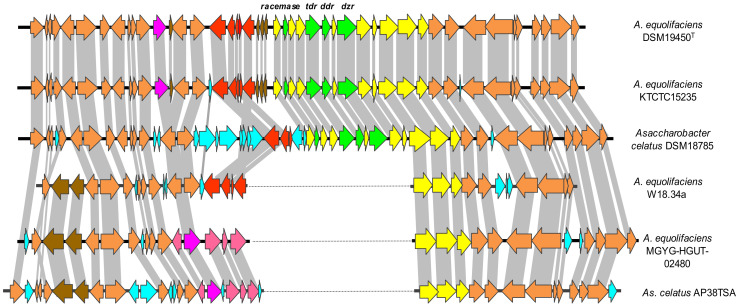
Diagram showing the genetic organization of upstream and downstream ORFs within the equol gene cluster in several strains of *A. equolifaciens* and *As. celatus*. In green, genes coding for well-known proteins involved in equol biosynthesis: racemase (*racemase*), daidzein reductase (*dzr*), dihydrogenistein reductase (*ddr*), and tetrahydrodaidzein reductase (*tdr*); in yellow, genes coding proteins with activity in daidzein metabolism; in light brown, conserved genes along all analyzed strains; in dark brown, red, pink, and purple, genes present in certain strains but not in others; and in pale blue, strain-specific genes. *A. equolifaciens* DSM19450^T^ and *As. celatus* DSM18785^T^ have been reported to be equol producers, while *A. equolifaciens* W18.34a does not produce equol.

In agreement with its equol-negative phenotype, no genes encoding reductases homologous to those involved in equol formation in *A. equolifaciens, As. celatus*, or *Lactococcus garvieae* were identified in the W18.34a genome. Comparison of DNA and deduced protein sequences from W18.34a to those of the equol-producing strain *A. equolifaciens* DSM19450^T^ (GCA_000478885.1) showed the former to lack a region of about 11 kbp (Figure 3). This region contains a major part of the equol operon of *A. equolifaciens* DSM19450^T^ [45]. In contrast, shared flanking ORFs upstream and downstream of the equol operon showed a deduced amino acid identity of 80–99% (Figure 3). Analysis of other genomes from NCBI showed that *A. equolifaciens* DSM19450^T^, *A. equolifaciens* KTCTC15235 (GCA_003428235.1), *As. celatus* DSM18785^T^ (GCA_003726015.1), and *As. celatus* JCM14811 (GCA_003428485.1) harbored a complete equol operon in their genomes, while W18.34a, *A. equolifaciens* ResAG-91 (GCA_009755265.1), *A. equolifaciens* MGYG-HGUT-02480 (GCA_902387565.1), *As. celatus* AP38TSA (GCA_003340305.1), and *As. celatus* OB21 GAM11 (GCA_003340325.1) did not. The genetic organization of upstream and downstream ORFs around the equol gene cluster in several strains is shown in Figure 4. Furthermore, the assembled genome sequence of an uncultured *Adlercreutzia* spp. strain from a metagenomic project (SRA accession ERS2710141; GCA_900542605.1) also lacked equol genes, while maintaining highly homologous flanking DNA sequences to those of the above strains.

## 4. Discussion

The high isoflavone consumption of Asian populations (compared to that of Westerners) has been epidemiologically associated with less severe menopause symptoms and a lower prevalence of cardiovascular diseases, osteoporosis, and cancer [1]. However, the actual metabolite(s) that impart these beneficial health effects, the target tissue(s), and the underlying signaling cascade(s) have yet to be discovered [46]. Although it is well established that the synthesis of equol from daidzein is carried out exclusively by certain members of the gut microbiota, the actual microbes involved are not well-identified [1,5,16]. In this work, three fecal samples, which have proven to produce equol [30], were used as a source of isoflavone-acting microbes.

Plating on GAM-Arg agar has been shown to be appropriate for isolating strains of the majority and subdominant bacterial populations from feces [47] including members of the class *Coriobacteriia*, family *Eggerthellaceae*, where most equol producers of intestinal origin are currently allocated [17]. The qPCR strategy targeting the *tdr* gene, however, did not result in the identification of any equol-producing isolate, even though 73 of them were initially considered as possibly positive. In agreement with their obligate anaerobic nature, most intestinal species possess large numbers of reductase-encoding genes [48]. As an example, 166 ORFs have been annotated as reductase-encoding genes in the genome of *A. equolifaciens* W18.34a [38]. Some of these might have regions of similarity to those of the *tdr* genes used in the design of the present degenerate primers, which led to unspecific amplification. If isolation of equol producers is the goal, a different approach and/or a higher testing effort would be required. As reported elsewhere [49], equol production might also result from the complementary activity of two or more microbes, which will complicate the identification of equol producers.

Daidzein and genistein were partially transformed by many isolates. For daidzein, small amounts of dihydrodaidzein and moderate amounts of *O*-DMA were quantified in the culture supernatants of several strains. The activity of one or more of the (unspecific) reductases above-mentioned might be responsible for the formation of small amounts of these isoflavone-derived metabolites. However, under the present study conditions, no strain tested produced equol. As the donor fecal cultures produced equol [30], the production of this compound was deemed feasible if strains from different species were combined in groups of two, three, or four, but this was not the case, which indicates that those tested had no complementary activities that would lead to equol synthesis. Similarly, moderate amounts of dihydrogenistein were detected in the supernatant of some strains when cultured with genistein (some of which did not produce dihydrodaidzein from daidzein). The control strain *S. isoflavoniconvertens* DSM 22006^T^ has been reported to be a 5-hydroxy equol producer [23]. However, the lack of an appropriate commercial standard hindered the determination in this study of this isoflavone-derivative. As previously reported and discussed, isoflavones may be transformed into a variety of unidentified metabolites [30,50]. In the absence of standards, identification of some of those that are known to be produced (e.g., 5-hydroxy-equol, 5-hydroxy-dehydroequol) may require high-performance liquid chromatography (HPLC) and gas chromatography/mass spectrometry (GC/MS) analysis and/or sophisticated chirality studies natural and chemically synthesized substances [51,52].

Apart from the deglycosilation step [8,53], our knowledge of the microbes and molecular pathways involved in isoflavone metabolism is still limited [54]. Most intestinal bacteria acting on isoflavones belong to the family *Eggerthellaceae* [17]. However, whether other bacterial species in the gut participate in the metabolism of isoflavones and the formation of equol and/or 5-hydroxy-equol remains unknown. In addition, the issue of whether the production of these compounds by the *Eggerthellaceae* is a family-, species-, or strain-specific trait is yet to be resolved [16]. It was therefore surprising to identify an *A. equolifaciens* strain from the feces of an equol-producing woman that did not produce equol. Genome analysis of this strain revealed a major part of the equol gene cluster to be absent. In the type strain of this species, *A. equolifaciens* DSM19450^T^, this cluster is composed of 10–13 ORFs organized into an operon-like structure [45]. Conceivably, the absence of equol-related genes is the cause of the equol non-producing phenotype in *A. equolifaciens* W18.34a. Analysis of the available genomes in the National Center for Biotechnology Information (NCBI) database identified more or less equal numbers of *A. equolifaciens* and *As. celatus* strains with and without most of the equol-related genes. All equol-producing strains harbor equol-associated genes, particularly those coding for a racemase and three reductases, namely daidzein reductase, dihydrodaidzein reductase, and tetrahydrodaidzein reductase [23,24,25,26,55,56,57]. However, with the exception of *A. equolifaciens* W18.34a, nothing is known about the equol phenotype of strains lacking genes within the equol operon. Whether there has been a deletion in strains lacking the locus or a gain-of-function in equol producers is currently a matter of speculation. However, the fact that in all strains lacking genes of the equol operon both upstream and downstream genes conserve a high degree of linearity and their deduced proteins show strong amino acid identity argues for the deletion of genes in certain strains. This suggests that the equol-producing phenotype does not currently provide a selective advantage in the human intestine to bacteria, thus leading to a loss of metabolic function. This agrees well with only a small percentage of humans (depending on dietary habits and human community) carrying equol-producing microbes in their gut [11,12,58], while all the animals tested so far are able to produce equol in response to soy or daidzein consumption [59]. The presence in the human gut of equol producing and equol non-producing *Eggerthellaceae* is further strengthened by the repeated counting of similar numbers of equol-related taxa in fecal samples from equol producers and non-producers [30,60,61,62,63]. As a consequence, determining the equol-producing status in humans based on taxonomic criteria alone is unreliable [63,64].

## 5. Conclusions

In this study, strains of several bacterial species from human feces able to produce small to moderate amounts of dihydrodaidzein and *O*-DMA from daidzein and of dihydrogenistein from genistein were detected. No association was seen between the formation of dihydrodaidzein from daidzein and that of dihydrogenistein from genistein, although some strains produced both isoflavone derivatives. None of the strains tested produced equol from daidzein, even though isolate W18.34a (IPLA 37004) was identified as *A. equolifaciens*. Other bacterium or a bacterial consortium not isolated in the present work may be responsible for the equol-producing phenotype of the women who supplied the fecal samples. Genome analyses of W18.34a suggested the deletion of most of the genes in the equol operon in this strain, and in others of *A. equolifaciens* and *As. celatus*. This argues in favor of the coexistence of equol-producing and non-producing bacterial strains in the human gut, suggesting the former phenotype does not provide a selective advantage. However, more studies are still required to unravel the complex relationships of isoflavones and components of the gut microbiota with emphasis on the synthesis of physiologically-active derived molecules.

## Figures and Tables

**Table 1:** Molecular identification by 16S rRNA gene sequencing of the isolates in this study.

Phylum	Class	Family	Species	Isolate ^a^	% Identity	Homology to Sequence
*Proteobacteria*	*γ-Proteobacteria*	*Enterobacteriaceae*	*Escherichia coli*	W3.16	98.76	DQ857002.1
			*E. coli*	W3.21	99.00	CP026491.1
			*E. coli*	W8.4b	100.00	KR265473.1
			*E. coli*	W8.5	99.00	MF067516.1
			*E. coli*	W8.22b	99.00	CP019455.1
			*E. coli*	W8.24b	99.00	CP026491.1
			*E. coli*	W8.35b	99.00	KR265473.1
			*E. coli*	W8.36b	99.31	MH782081.1
			*E. coli*	W8.39a	99.68	KY524291.1
			*E. coli*	W8.40	99.79	KY524291.1
			*E. coli*	W8.47	99.00	CP019455.1
			*E. coli*	W8.52b	99.00	KR265473.1
			*E. coli*	W8.53b	99.00	KP789326.1
			*E. coli*	W8.56c	98.41	KP244257.1
			*E. coli*	W18.17b	99.88	MH111527.1
			*E. coli*	W18.34b	99.00	CP019455.1
			*E. coli*	W18.19b	99.00	LC270191.1
*Firmicutes*	*Bacilli*	*Enterococcaceae*	*Enterococcus durans*	W8.29	99.21	KY962884.1
			*Enterococcus lactis*	W8.56b	99.68	MH431810.1
	*Clostridia*		*Anaerococcus* spp.	W3.10b	96.69	NR_114314.1
			*Anaerococcus* spp.	W8.44a	95.15	NR_114314.1
			*Anaerococcus vaginalis*	W3.18	98.43	NR_114314.1
			*A. vaginalis*	W8.44b	98.33	NR_041937.1
		*Ruminococcaceae*	*Bittarella massiliensis*	W18.24a	99.46	LN881596.1
			*B. massiliensis*	W18.24b	99.55	LN881596.1
			*Finegoldia magna*	W8.4a	99.80	KC311751.1
			*F. magna*	W8.35a	99.00	KR232883.1
			*Peptoniphilus gorbachii*	W8.22a	99.00	NR_115885.1
			*P. gorbachii*	W8.33	99.00	NR_115885.1
		*Streptococcaceae*	*Streptococcus anginosus*	W8.21	99.00	JN787165.1
*Bacteroidetes*	*Bacteroidia*	*Bacteroidaceae*	*Bacteroides vulgatus*	W8.28	99.00	CP013020.1
		*Porphyromonadaceae*	*Parabacteroides* spp.	W8.41	95.56	CP022754.1
*Actinobacteria*	*Actinomycetales*	*Actinomycetaceae*	*Actinomyces neuii*	W8.24a	99.56	NR_042429.1
	*Bifidobacteriales*	*Bifidobacteriaceae*	*Bifidobacterium adolescentis*	W8.34	99.30	KC174855.1
			*B. adolescentis*	W8.36a	99.00	KC174855.1
			*B. adolescentis*	W8.39b	99.00	LT223639.1
			*B. adolescentis*	W8.45	99.00	LT223639.1
			*B. adolescentis*	W8.54b	96.31	HM009032.1
			*Bifidobacterium animalis*	W3.3	99.31	MK561779.1
			*Bifidobacterium longum*	W8.17	99.00	KC174855.1
			*B. longum*	W8.49a	98.00	HM009032.1
	*Coriobacteriia*	*Coriobacteriaceae*	*Collinsella aerofaciens*	W8.23	99.00	KP233323.1
			*C. aerofaciens*	W8.49b	99.00	KR232866.1
			*Collinsella massiliensis*	W18.17a	100.00	NR_144579.1
			*C. massiliensis*	W18.9	99.00	NR_144579.1
			*C. massiliensis*	W18.21a	99.00	NR_144579.1
			*C. massiliensis*	W18.21c	99.00	NR_144579.1
			*C. massiliensis*	W18.21d	99.00	NR_144579.1
			*C. massiliensis*	W18.25	99.00	NR_144579.1
			*C. massiliensis*	W18.29	99.00	NR_144579.1
			*Collinsella stercoris*	W18.1	97.80	KP233278.1
	*Eggerthellaceae*		*Adlercreutzia equolifaciens*	W18.34a	100.00	AB693938.1
			*Gordonibacter urolithinfaciens*	W18.23	99.00	NR_148261.1
			*G. urolithinfaciens*	W18.26	100.00	LT900217.1
			*Eggerthella lenta*	W3.2	99.00	KX683977.1
			*E. lenta*	W3.10a	99.00	KX683993.1
			*E. lenta*	W3.11	99.00	KX683977.1
			*E. lenta*	W3.12	99.00	KX683977.1
			*E. lenta*	W3.13	100.00	KX683977.1
			*E. lenta*	W3.14	99.00	KX683977.1
			*E. lenta*	W3.15	100.00	KX683977.1
			*E. lenta*	W8.15	98.00	KX683885.1
			*E. lenta*	W8.16	99.00	KX683992.1
			*E. lenta*	W8.27	99.00	KP944189.1
			*E. lenta*	W8.31	99.00	KP944189.1
			*E. lenta*	W8.42	99.00	JX104026.1
			*E. lenta*	W8.43	99.00	KX683992.1
			*E. lenta*	W8.52a	99.00	KP944190.1
			*E. lenta*	W8.53a	99.00	KX683992.1
			*E. lenta*	W8.54a	99.00	KP944189.1
			*E. lenta*	W8.56a	98.00	JX104026.1
			*E. lenta*	W18.18	99.00	KX683993.1
			*E. lenta*	W18.21b	100.00	KX683993.1

^a^ Isolates were recovered from fresh stool samples of three women (W3, W8, and W18). In cultures, these fecal samples had been shown to produce dihydrodaidzein and equol from daidzein and dihydrogenistein from genistein [30].

**Table 2:** Production of equol from daidzein by type strains of equol-producing species.

Equol-Producing Strain	Daidzein Supplementation (µM)															
	12.5				25				50				100			
	Daidzein-Derived Metabolite (µM)															
	Daidzein		Equol		Daidzein		Equol		Daidzein		Equol		Daidzein		Equol	
	24 h	48 h	24 h	48 h	24 h	48 h	24 h	48 h	24 h	48 h	24 h	48 h	24 h	48 h	24 h	48 h
*A. equolifaciens* DSM19450^T^	-	-	16.6	7.7	-	-	33.6	13.5	-	-	60.9	25.8	2.3 *	-	126.2	53.4
*As. celatus* DSM18785^T^	-	-	3.1	3.3	nd	-	nd	6.3	-	-	14.6	12.0	-	-	27.7	27.8
*E. mucosicola* DSM19490^T^	2.1 *	1.4 *	4.5	2.0 *	-	-	17.9	6.7	5.1 *	1.4 *	26.2	10.5	60.3	36.2	nd	nd
*S. equolifaciens* DSM24851^T^	-	-	5.8	7.4	-	-	14.4	13.3	1.1 *	-	39.2	23.3	1.8 *	-	110.0	47.8
*S. isoflavoniconvertens* DSM22006^T^	-	-	18.2	7.9	-	-	37.0	14.3	-	-	67.6	24.6	-	-	149.0	55.0

-, not detected; nd, not done; asterisks denote samples with a concentration of the compounds below the LoQ.

**Table 3:** Metabolism of daidzein and genistein by isolates and controls and the metabolites detected.

	Isolate	DZEN ^a^ (100 μM)				GTEN (100 μM)	
		DZEN	D-DZEN	*O*-DMA	EQUOL	GTEN	D-GTEN
*Escherichia coli*	W3.16	61.2	-	-	-	64.9	4.8 *
*E. coli*	W3.21	61.9	-	-	-	60.0	4.3 *
*E. coli*	W8.4b	65.8	-	-	-	60.6	4.2 *
*E. coli*	W8.24b	85.1	-	3.3 *	-	65.9	4.2 *
*E. coli*	W8.35b	76.3	-	2.8 *	-	60.4	5.5 *
*E. coli*	W8.36b	73.2	1.5 *	6.8	-	68.6	-
*E. coli*	W8.40	73.2	3.0 *	3.0 *	-	66.6	-
*E. coli*	W8.47	83.8	-	5.5 *	-	62.8	-
*E. coli*	W8.53b	82.5	-	10.3	-	72.1	-
*E. coli*	W18.17b	87.5	-	11.2	-	72.0	-
*Enterococcus durans*	W8.29	85.1	-	3.7 *	-	117.9	-
*Enterococcus lactis*	W8.56b	87.0	-	10.8	-	71.7	-
*Anaerococcus* spp.	W3.10b	93.6	-	10.9	-	75.5	-
*Anaerococcus* spp.	W8.44a	82.1	3.4	2.9 *	-	62.9	-
*Anaerococcus vaginalis*	W3.18	62.0	-	2.3 *	-	78.9	-
*Anaerococcus vaginalis*	W8.44b	79.8	-	-	-	48.2	-
*Bittarella massiliensis*	W18.24a	84.5	-	10.2	-	78.2	-
*Finegoldia magna*	W8.4a	61.2	-	-	-	55.7	2.7 *
*F. magna*	W8.35a	68.0	1.1 *	5.2 *	-	62.6	-
*Peptoniphilus gorbachii*	W8.22a	69.5	3.9	10.0	-	40.3	4.3 *
*P. gorbachii*	W8.33	59.6	2.9 *	2.5 *	-	40.3	-
*Streptococcus anginosus*	W8.21	99.6	-	2.9 *	-	53.7	-
*Bacteroides vulgatus*	W8.28	73.1	-	3.7 *	-	92.3	6.3
*Parabacteroides* spp.	W8.41	72.3	-	-	-	68.6	-
*Actinomyces neuii*	W8.24a	53.5	2.8 *	5.1 *	-	83.2	7.7
*Bifidobacterium adolescentis*	W8.34	96.4	-	-	-	79.8	16.0
*B. adolescentis*	W8.36a	110.5	-	3.4 *	-	109.8	8.3
*B. adolescentis*	W8.45	86.6	-	5.3	-	123.1	10.5
*Bifidobacterium animalis*	W3.3	85.2	-	10.4	-	88.8	-
*Bifidobacterium longum*	W8.17	62.2	3.8	10.3	-	82.2	3.9 *
*B. longum*	W8.49a	136.1	-	2.3 *	-	124.4	-
*Collinsella aerofaciens*	W8.23	109.1	3.7	11.7	-	84.6	6.1 *
*C. aerofaciens*	W8.49b	70.9	-	-	-	63.2	-
*Collinsella massiliensis*	W18.9	62.3	-	12.0	-	52.3	-
*C. massiliensis*	W18.17a	85.7	-	11.2	-	69.5	-
*C. massiliensis*	W18.21a	91.8	-	8.3	-	81.7	-
*C. massiliensis*	W18.21c	89.2	-	8.8	-	70.9	-
*C. massiliensis*	W18.21d	85.5	-	10.4	-	79.9	-
*C. massiliensis*	W18.25	84.6	-	12.0	-	76.5	-
*C. massiliensis*	W18.29	78.4	-	-	-	93.6	-
*Collinsella stercoris*	W18.1	87.8	-	9.4	-	81.2	-
*Adlercreutzia equolifaciens*	W18.34a	87.4	-	11.6	-	71.5	-
*Gordonibacter urolithinfaciens*	W18.23	76.9	-	10.8	-	73.3	-
*Eggerthella lenta*	W3.2	90.7	-	12.5	-	95.1	-
*E. lenta*	W3.10a	86.4	-	11.3	-	78.0	-
*E. lenta*	W3.11	89.1	-	11.6	-	80.4	-
*E. lenta*	W3.12	86.4	-	8.7	-	79.1	-
*E. lenta*	W3.13	88.3	-	10.7	-	81.9	-
*E. lenta*	W3.14	89.7	-	11.6	-	79.3	-
*E. lenta*	W8.15	66.3	3.9	9.8	-	58.6	6.4
*E. lenta*	W8.16	65.7	3.3	8.7	-	58.4	3.4 *
*E. lenta*	W8.27	94.3	-	3.7 *	-	82.1	4.4 *
*E. lenta*	W8.31	117.2	-	-	-	90.0	6.8
*E. lenta*	W8.42	78.1	3.5	2.9 *	-	62.2	-
*E. lenta*	W8.52a	97.9	-	8.9	-	63.5	-
*E. lenta*	W8.53a	87.3	-	9.0	-	73.0	-
*E. lenta*	W18.18	74.1	-	12.3	-	66.2	-
Uninoculated medium	-	98.1 ± 5.8	1.4 ± 0.2	-	-	95.6 ± 0.4	1.6 ± 0.6
*S. isoflavoniconvertens*	DSM 22006^T^	-	1.6 ± 0.2	-	164.3 ± 13.4	55.3	19.0

^a^ DZEN, daidzein; GTEN, genistein; D-DZEN y D-GTEN, dihydrodaidzein and dihydrogenistein, respectively; *O*-DMA, *O*-desmethylangolensin; -, not detected; asterisks indicate cultures with a concentration of the compound below the LoQ.

## Supplementary Materials

The following are available online at https://www.mdpi.com/2218-273X/10/6/950/s1, Figure S1: Dendogram of similarity of the combined typing profiles obtained with primers OPA18, M13, and BoxA2R expressed by the Simple Matching (SM) coefficient. Clustering was performed by the unweighted pair group method using arithmetic averages (UPGMA). The dotted line indicates the repeatability of the combined typing method (~94%).
