# Supplementary material for: Metabolism of Soy Isoflavones by Intestinal Bacteria: Genome Analysis of an Adlercreutzia equolifaciens Strain That Does Not Produce Equol
Source: Biomolecules. 2020 Jun 23;10(6):950. doi: 10.3390/biom10060950 (PMC7355428; doi:10.3390/biom10060950)
Supplement: Supplementary file 1 [file biomolecules-10-00950-s001.pdf]

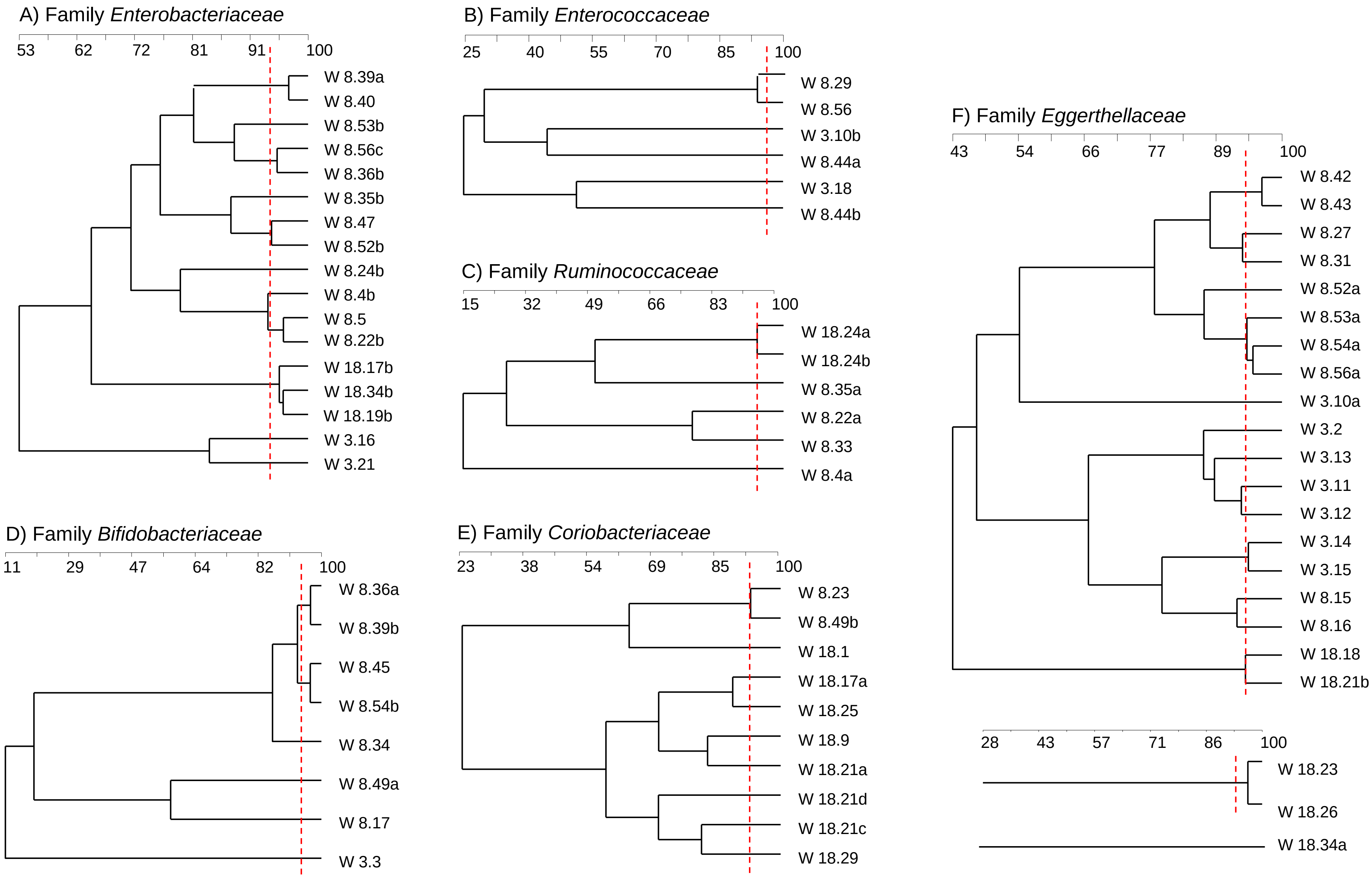

Figure S1.- Dendogram of similarity of the combined typing profiles obtained with primers OPA18, M13, and BoxA2R expressed by the Simple Matching (SM) coefficient. Clustering was performed by the unweighted pair group method using arithmetic averages (UPGMA). The dotted line indicates the repeatability of the combined typing method (~94%).
